# Oocyte Aging as a Systems-Level Failure of Cellular Quality Control Networks: Insights from Comparative Biology

**DOI:** 10.3390/biology15141143

**Published:** 2026-07-14

**Authors:** Magda Sochaczewska, Waclaw Tworzydlo

**Affiliations:** 1 Department of Developmental Biology and Invertebrate Morphology, Institute of Zoology and Biomedical Research, Faculty of Biology, Jagiellonian University, Gronostajowa 9, 30-387 Krakow, Poland; magda.sochaczewska@doctoral.uj.edu.pl; 2 Doctoral School of Exact and Natural Sciences, Jagiellonian University, Lojasiewicza 11, 30-348 Krakow, Poland

**Keywords:** oocyte aging, reproductive aging, biomolecular condensates, mitochondrial quality control, mitophagy, proteostasis, epigenetic drift, germline aging

## Abstract

Oocyte aging is a conserved biological process that limits reproductive success across animal taxa. While most research has focused on mammalian systems, particularly humans, comparative studies reveal both shared molecular mechanisms and lineage-specific adaptations that shape oocyte longevity. Here, we synthesise current knowledge on chromosomal instability, mitochondrial dysfunction, proteostasis collapse and epigenetic alterations, integrating insights from insects, nematodes and vertebrates. We argue that these phenomena should not be viewed as independent hallmarks of reproductive aging but rather as interconnected manifestations of a progressive decline in cellular quality control networks. By comparing taxa, we identify both conserved vulnerabilities, including cohesin loss, mitochondrial deterioration and genome instability, and lineage-specific adaptations that mitigate their effects. We further propose the hypothesis that biomolecular condensates, including the Balbiani body, nuage and recently described endolysosomal assemblies, may function as higher-order organizational hubs coordinating mitochondrial quality control, proteostasis and genome defence. This perspective provides a unifying framework for understanding oocyte aging as a systems-level process and highlights new directions for future studies of reproductive longevity.

## Simple Summary

Female fertility declines with age largely because oocytes progressively lose their ability to support normal fertilization and embryonic development. Although most studies of oocyte aging have focused on humans, many of the underlying mechanisms are shared across animal groups. In this review, we compare current knowledge from vertebrate and invertebrate models to identify both conserved and species-specific processes involved in oocyte aging. We discuss how chromosome instability, mitochondrial dysfunction, impaired protein quality control and epigenetic changes contribute to the gradual decline of oocyte quality. We also highlight the emerging role of biomolecular condensates, specialized cellular compartments and propose the hypothesis that they may coordinate multiple quality control pathways. By integrating evidence from diverse animal systems, we suggest that oocyte aging is best understood as a progressive failure of interconnected cellular maintenance mechanisms rather than the consequence of a single defect. Understanding how these systems interact may improve our knowledge of reproductive aging and provide new directions for preserving fertility and studying the biology of aging more broadly.

## 1. Introduction

The age-related decline in female fertility is a well-recognized biological phenomenon, driven largely by progressive reductions in oocyte quality and quantity. Oocyte aging directly affects fertilization success, embryonic development and offspring viability, and is associated with increased rates of miscarriage and chromosomal abnormalities in many animal species [1,2]. It is useful to distinguish between two biologically distinct forms of aging: reproductive (chronological) aging, which takes place during the prolonged lifespan of oocytes within the ovary, and postovulatory aging, which occurs after ovulation. Although these clearly disparate processes differ in timescale and underlying triggers, both alter regulatory networks that maintain oocyte competence [1,2].

Much of our current understanding of oocyte aging comes from studies of mammals, particularly humans. However, this perspective neglects the remarkable evolutionary diversity of mechanisms that regulate oocyte maintenance. Insects, nematodes and other organisms provide experimentally tractable systems in which reproductive aging occurs over compressed timescales or is shaped by unique ecological and life-history strategies. Comparative studies offer an opportunity to identify both conserved principles and lineage-specific adaptations governing oocyte longevity.

For example, *Drosophila melanogaster* has emerged as a powerful model for investigating the molecular basis of oocyte aging. Age-related changes in oocyte quality can be observed within days and include increased meiotic errors, chromosomal instability and impaired mitochondrial function [3,4]. Social insects provide a contrasting perspective, as reproductive queens may maintain fertility for years through enhanced antioxidant protection and mechanisms promoting genome stability [5,6]. Beyond insects, naturally long-lived vertebrates are also valuable comparative models for understanding reproductive aging. The naked mole-rat (*Heterocephalus glaber*), an exceptionally long-lived eusocial mammal, maintains prolonged ovarian function and exhibits postnatal germ-cell proliferation, challenging the conventional view of a fixed ovarian reserve and progressive reproductive decline [7,8]. Such exceptional species may reveal evolutionary adaptations that preserve oocyte quality and reproductive longevity over extended lifespans. In *Caenorhabditis elegans*, oocyte quality is maintained through extensive germline apoptosis and efficient mitochondrial quality control, highlighting alternative evolutionary solutions to the challenge of reproductive aging [9,10].

In addition to intrinsic cellular processes, oocyte quality is strongly influenced by the surrounding ovarian microenvironment. Supporting cells, including granulosa cells in mammals and nurse cells and follicular cells in insects, provide nutrients, molecular signals and quality control functions that are essential for maintaining oocyte integrity [11,12,13]. Age-related deterioration of these interactions further contributes to reproductive decline.

Oocytes are among the longest-lived cells in the animal body. They are typically formed early in life and may remain in a state of meiotic arrest for months, years or even decades, depending on the species. This prolonged quiescence exposes them to the cumulative effects of genetic, metabolic and environmental stress, making the preservation of cellular integrity a major biological challenge.

This review was prepared as a narrative synthesis of the current literature on oocyte aging. Particular emphasis was placed on comparative studies involving both vertebrate and invertebrate model organisms. The literature was identified primarily through searches of the PubMed and Web of Science databases using combinations of keywords including *oocyte aging*, *oogenesis*, *mitochondria*, *mitophagy*, *proteostasis*, *epigenetics*, *biomolecular condensates*, *Balbiani body*, *nuage*, *germ plasm*, *insects*, and *comparative biology*. As this is a narrative rather than a systematic review, no formal publication date restrictions or predefined inclusion/exclusion criteria were applied. Instead, priority was given to peer-reviewed studies providing mechanistic insights into oocyte aging, while seminal earlier publications were included where necessary to provide proper context.

In this review, we move beyond descriptive comparisons and instead ask three key questions:

1. Which mechanisms of oocyte aging are evolutionarily conserved?
2. Which mechanisms are lineage-specific adaptations?
3. What do these differences reveal about fundamental constraints on germline longevity?

We argue that oocyte aging is best understood not as the consequence of a single dominant defect but as a systems-level failure of interacting quality control networks. We further propose the hypothesis that biomolecular condensates may act as higher-order organizational hubs coordinating mitochondrial quality control, proteostasis, and genome defence providing a unifying framework for understanding reproductive aging within various animal taxa.

## 2. Molecular and Cellular Mechanisms of Oocyte Aging

### 2.1. Chromosomal and Meiotic Instability

**Figure 1:**
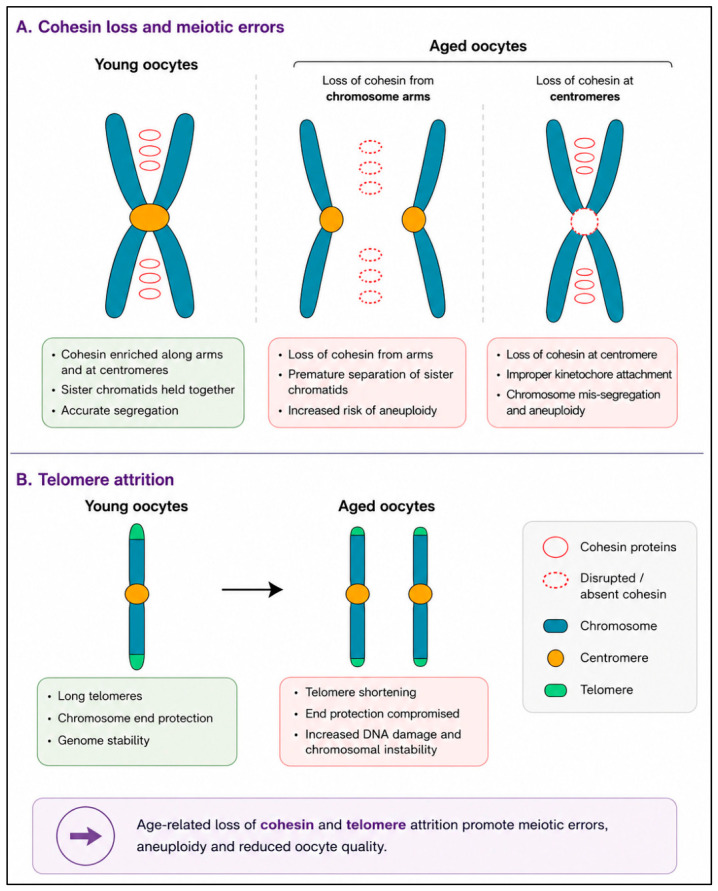
Chromosomal and meiotic instability during oocyte aging. (**A**) Age-dependent deterioration of cohesin complexes, chromosome segregation machinery and (**B**) telomere maintenance promotes genome instability, aneuploidy and declining oocyte quality.

Aged oocytes across diverse animal taxa exhibit a pronounced increase in chromosomal instability and aneuploidy, although the underlying mechanisms and biological consequences vary among lineages. In mammals, including humans, this instability is primarily attributed to the age-related deterioration of the cohesin complex (e.g., REC8) and of its centromeric protector SGO2/shugoshin, which are essential for maintaining sister chromatid cohesion during prolonged meiotic arrest. Their gradual loss promotes premature separation of sister chromatids, chromosome misalignment and segregation errors during meiotic divisions [14,15,16]. Age-associated weakening of the spindle assembly checkpoint, together with mitochondrial dysfunction, oxidative stress and reduced ATP availability, is thought to impair spindle assembly and chromosome segregation, thereby increasing the risk of meiotic errors [17,18] (Figure 1A).

Comparative studies in insects, particularly *D. melanogaster*, indicate that similar mechanisms operate over much shorter timescales. In *D. melanogaster*, age-related reduction in cohesin components such as SMC1 leads to increased nondisjunction, premature chromatid separation and spindle instability within days of oocyte aging [4]. These findings closely parallel mammalian oocyte aging and highlight the value of insects as models for dissecting conserved mechanisms of meiotic failure.

Telomere dynamics represent another component of age-related genome instability (Figure 1B), although their role differs among taxa. In mammalian oocytes, telomere shortening has been associated with reduced developmental competence and lower oocyte quality, probably through increased vulnerability to oxidative stress and impaired chromosome end protection [19]. However, comparative data indicate that telomere shortening is not a universal feature of reproductive aging. In some insects, including diapause-associated bee species, telomere length may remain stable or even increase during developmental arrest, suggesting that telomere maintenance can be modified by life-history strategy and reproductive mode [6].

Social insects provide a particularly striking example of evolutionary adaptation in germline maintenance. Honeybee and bumblebee queens can remain reproductively active for years, despite the generally short reproductive lifespan of most insects. Their exceptional reproductive longevity has been linked to enhanced antioxidant protection, vitellogenin-mediated stress resistance and increased activity of pathways involved in chromosome maintenance and telomere regulation [5,20,21]. These caste-specific mechanisms illustrate how reproductive aging can be slowed or buffered by physiological adaptations.

In *C. elegans*, reproductive aging is also associated with impaired chromosome stability, accumulation of DNA damage and reduced efficiency of homologous recombination repair. Aged oocytes show defects in RAD-51-dependent repair and increased susceptibility to chromosome segregation errors. Conserved signalling pathways, including MAPK and insulin/IGF signalling, modulate this decline, while interventions affecting these pathways can partially preserve oocyte quality and reproductive lifespan [22,23].

### 2.2. Mitochondrial Homeostasis and Quality Control During Oocyte Aging

**Figure 2:**
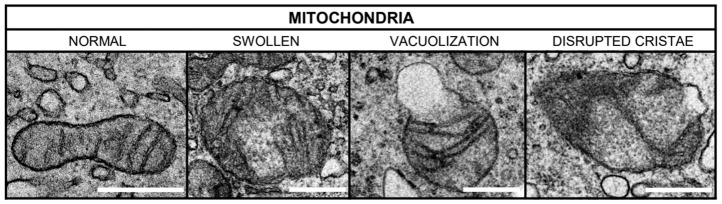
Ultrastructural changes in mitochondria during oocyte aging. Representative transmission electron micrographs illustrating age-related mitochondrial degeneration in oocytes obtained from aged females of a bush-cricket, *Roeseliana roeselii*. All transmission electron micrographs shown in this figure are original images obtained by the authors from aged oocytes of *Roeseliana roeselii*. Old females (at the end of the reproductive cycle) were collected in Kraków (southern Poland) and their ovaries were processed for transmission electron microscopy using glutaraldehyde/formaldehyde fixation followed by osmium tetroxide/potassium ferrocyanide postfixation. Scale bars: 500 nm.

Chromosomal instability has traditionally been considered a major determinant of oocyte quality; however, increasing evidence highlights mitochondrial dysfunction as a central integrative hub linking multiple aging pathways. According to the mitochondrial theory of aging, progressive accumulation of mitochondrial damage contributes to cellular senescence and functional decline. Mitochondria are among the most abundant organelles in oocytes and are essential for ATP production, calcium homeostasis and regulation of apoptosis. As oocytes age, mitochondrial number and quality decline, frequently accompanied by swelling, vacuolization and disruption of cristae architecture (Figure 2). To complement the literature review with representative ultrastructural examples, we include representative transmission electron micrographs obtained from our original observations of aged oocytes of the bush-cricket *Roeseliana roeselii* (Figure 2).

Mitochondrial DNA (mtDNA) is particularly vulnerable to damage because oxidative phosphorylation generates reactive oxygen species (ROS), which can induce oxidative stress. Aging is therefore associated with the accumulation of mtDNA mutations, impaired mitochondrial homeostasis and increased mitochondria-dependent apoptosis [24]. The resulting decline in ATP production and increase in ROS levels contribute to meiotic defects, chromosomal instability and reduced developmental competence.

**Figure 3:**
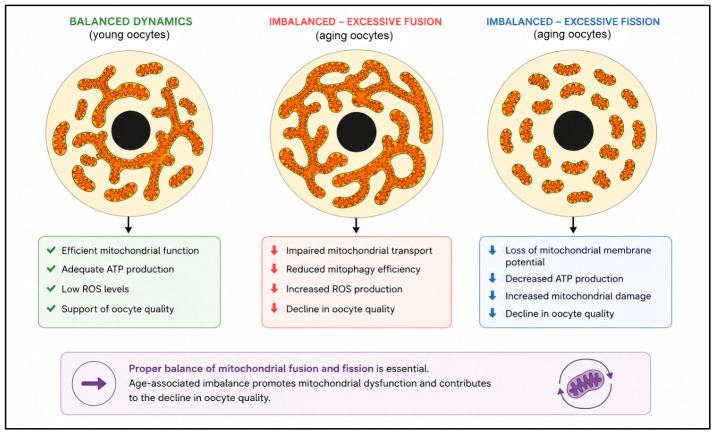
Mitochondrial homeostasis during oocyte aging. Maintenance of mitochondrial function depends on a balance between fusion, fission and quality control mechanisms. Age-related disruption of this balance contributes to mitochondrial dysfunction and reduced oocyte competence.

To preserve mitochondrial function throughout oogenesis, oocytes employ several complementary qualitycontrol mechanisms. Mitochondrial dynamics, including fusion and fission, play an important role in maintaining functional mitochondrial networks and facilitating the segregation of damaged organelles. Aging is frequently associated with disruption of this balance (Figure 3), often favouring mitochondrial fragmentation and progressive functional decline [25,26].

Because mitochondria are inherited almost exclusively through the maternal lineage, efficient mitochondrial quality control during oogenesis is essential to minimize the transmission of deleterious mtDNA mutations to the next generation. Several complementary mechanisms contribute to this process, including stochastic segregation of mitochondria during germline proliferation, selective elimination of dysfunctional organelles through mitophagy, preferential expansion of functional mitochondrial populations and apoptosis of severely affected germ cells [27,28,29]. These processes collectively reduce mutational load and help preserve a healthy mitochondrial population.

**Figure 4:**
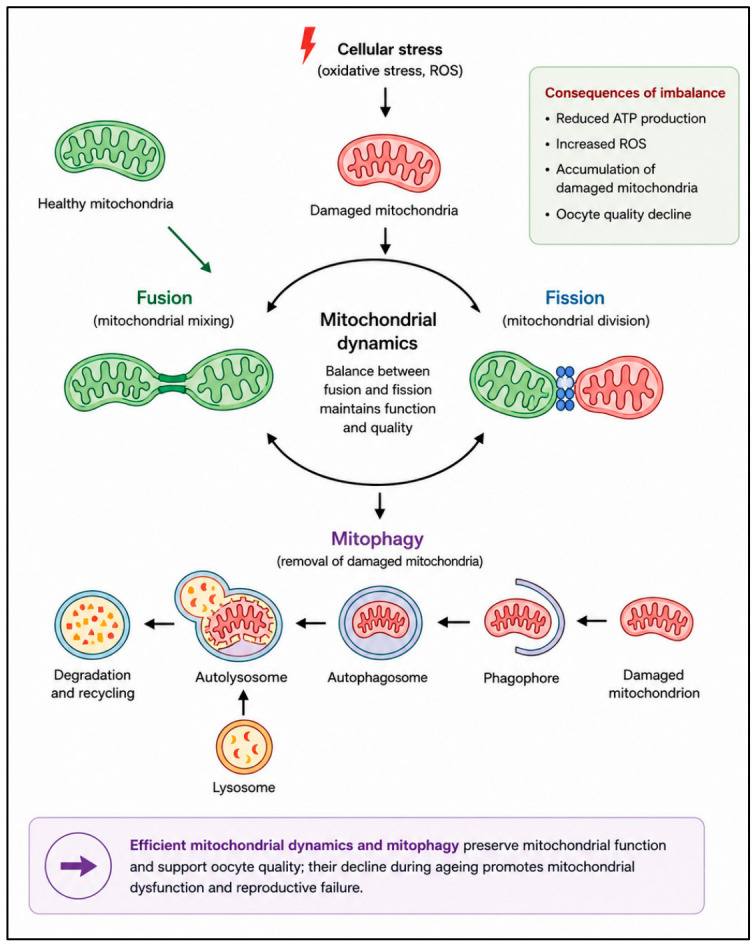
Mitophagy-mediated mitochondrial quality control in oocytes. Damaged mitochondria are selectively removed through mitophagy, limiting oxidative stress and preventing the accumulation of dysfunctional organelles during oogenesis.

Among these mechanisms, mitophagy represents a major pathway responsible for the selective removal of damaged or dysfunctional mitochondria (Figure 4). In young oocytes, mitophagy efficiently eliminates mitochondria that have lost membrane potential or accumulated oxidative damage, thereby maintaining the mitochondrial population required for ATP production, redox homeostasis and normal meiotic progression [30,31]. Increasing evidence suggests that mitophagic activity declines with age. Reduced mitochondrial clearance may lead to the accumulation of dysfunctional organelles, elevated ROS production and progressive deterioration of mitochondrial function. Consequently, mitochondrial DNA damage accumulates, ATP production decreases and oocyte developmental competence is reduced [32]. These observations suggest that impaired mitophagy may contribute substantially to reproductive aging rather than merely representing a downstream consequence of mitochondrial dysfunction.

Recent studies have identified an additional mechanism that may contribute to long-term mitochondrial preservation. Early oocytes of mammals and amphibians remodel their electron transport chain through the elimination of complex I, one of the major cellular sources of ROS, thereby reducing oxidative damage while maintaining metabolic activity [33]. Whether similar adaptations occur in invertebrate oocytes remains unknown.

Comparative studies further indicate that mitochondrial quality control extends beyond the oocyte itself. In insects, age-dependent accumulation of ROS in nurse cells and the selective transfer of high-quality organelles to the oocyte suggest that mitochondrial homeostasis is coordinated at the level of the entire germline cyst.

### 2.3. Protein Homeostasis Collapse

The maintenance of protein homeostasis (proteostasis) is essential for preserving oocyte quality throughout the reproductive lifespan. Proteostasis encompasses the synthesis, folding, trafficking and degradation of proteins, ensuring that damaged or misfolded proteins do not accumulate within the cell. As oocytes age, the efficiency of these quality control mechanisms progressively declines, resulting in the accumulation of protein aggregates, increased proteotoxic stress and reduced developmental competence [34]. Recent studies in mammalian oocytes have identified specialised endolysosomal assemblies, termed endolysosomal vesicular assemblies (ELVAs) [35] and endosomal-lysosomal organellar assemblies (ELYSAs) [36], which participate in the sequestration and degradation of damaged proteins. These structures act as dedicated quality control compartments that limit the harmful effects of protein aggregation and facilitate the lysosomal removal of damaged cellular components. Similar quality control compartments have recently been described in insect oocytes. In derived dermapterans (Eudermaptera), lysosome-like bodies accumulate at the posterior pole of the oocyte, forming a distinct membraneless structure termed the posterior pole lysosomal compartment (PPLC) [37]. This compartment exhibits several functional and structural similarities to mammalian ELVAs/ELYSAs, suggesting that these evolutionarily distant structures may represent convergent solutions for the sequestration and degradation of damaged cellular components, thereby contributing to long-term maintenance of oocyte quality. Notably, PPLC appears to be currently known only from the Eudermaptera and is absent in basal dermapteran lineages, suggesting that it represents an evolutionary innovation associated with derived earwig oogenesis [38].

In *C. elegans*, proteostasis is supported by stress-responsive chaperones such as UBL-5, which participate in the mitochondrial unfolded protein response and help maintain protein quality under conditions of cellular stress [39]. Age-dependent alterations in the localization and activity of these proteins reflect the progressive decline in proteome stability associated with reproductive aging.

The collapse of proteostasis has consequences that extend beyond protein aggregation itself. Impaired protein quality control can exacerbate mitochondrial dysfunction, increase oxidative stress and compromise other cellular maintenance pathways.

Current evidence suggests that age-related proteostasis failure contributes substantially to reproductive aging. Moreover, the emerging association between proteostasis pathways and biomolecular condensates raises the possibility that age-dependent alterations in condensate dynamics may represent an upstream event linking protein aggregation, mitochondrial dysfunction and the progressive decline of oocyte quality.

### 2.4. Biomolecular Condensates in Oocyte Aging

Biomolecular condensates represent a higher-order regulatory layer that coordinates mitochondrial quality control, RNA metabolism and proteostasis in oocytes. Their age-dependent dysfunction may therefore propagate defects across multiple interconnected quality control systems and contribute to the progressive decline in oocyte quality.

Developing oocytes of nearly all animal species contain a large organelle assemblage referred to as the Balbiani body [40,41]. Despite considerable morphological diversity among taxa, the Balbiani body is typically composed of numerous mitochondria associated with electron-dense granulo-fibrillar material known as nuage. Recent studies have demonstrated that the Balbiani body is a large biomolecular condensate formed through liquid–liquid phase separation (LLPS), driven by intrinsically disordered proteins such as Bucky ball (Buc) [42]. During oogenesis, this condensate undergoes a transition from a liquid-like to a more solid-like state, suggesting dynamic regulation of its physical properties.

The Balbiani body has long been associated with the spatial organization of organelles and maternal determinants, but accumulating evidence suggests that it also plays an important role in mitochondrial quality control. Mitochondria within the Balbiani body form highly interconnected networks characterised by elevated membrane potential compared with mitochondria dispersed throughout the ooplasm [43]. These observations support the hypothesis that the Balbiani body participates in the selective retention of functional mitochondria and the elimination of defective organelles before their transmission to the next generation. Whether aging alters the architecture, material properties or selective functions of the Balbiani body remains largely unexplored.

Another evolutionary conserved biomolecular condensate present in germ cells is the nuage. This non-membrane-bound structure is enriched in RNA-processing proteins and components of the piRNA pathway, playing crucial roles in RNA metabolism, transposon silencing and maintenance of genome stability [44,45]. Increasing evidence indicates that age-dependent deterioration of nuage function contributes to impaired RNA regulation, defective DNA repair and loss of germline integrity. In *C. elegans*, disruption of nuage-associated pathways correlates with reduced oocyte quality, increased apoptosis and progressive reproductive decline [46].

Importantly, biomolecular condensates are increasingly recognised as dynamic structures whose biological activity depends on their physical properties, including liquidity, viscosity and molecular exchange rates. Studies in other aging systems have demonstrated that age-related changes in condensate dynamics can impair their function and promote the accumulation of damaged proteins and nucleic acids. It is therefore plausible that similar processes occur in aging oocytes, affecting the ability of condensates to coordinate mitochondrial maintenance, RNA processing and genome protection.

**Figure 5:**
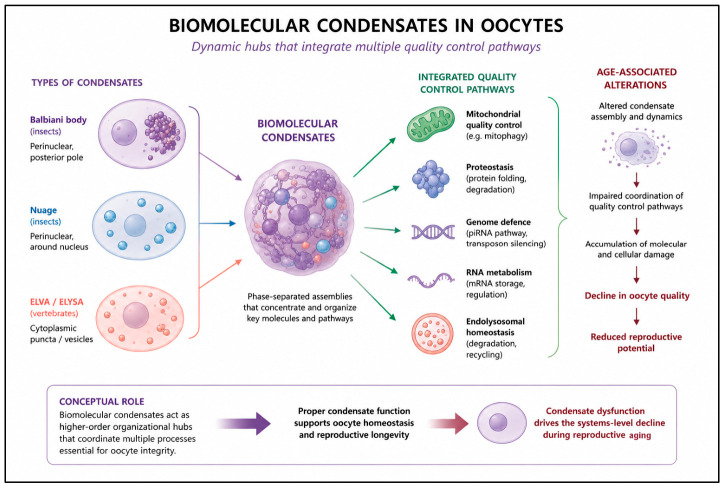
Proposed hypothesis that biomolecular condensates may act as regulators of oocyte homeostasis. The Balbiani body, nuage and endolysosomal assemblies function as dynamic condensate-based compartments involved in mitochondrial quality control, RNA metabolism, proteostasis and genome defence. Proposed age-related alterations in condensate physiology may lead to reduced oocyte quality and as a consequence to reduced reproductive potential.

This concept gains additional support from discoveries of endolysosomal assemblies in mammalian oocytes, including ELVAs and ELYSAs [35,36] as well as PPLC in dermapterans [37] (see above) which appear to share several features with phase-separated condensates, e.g., the absence of a limiting membrane and positive staining with Proteostat dye [34,35,36,37]. Together with the Balbiani body and nuage, these structures may constitute an interconnected network of condensate-based compartments responsible for integrating mitochondrial quality control, proteostasis and RNA regulation (Figure 5).

Collectively, these observations suggest that biomolecular condensates may represent central organizational hubs within the oocyte (Figure 5). Rather than functioning as isolated structures, they may coordinate multiple quality control pathways. Based on the available evidence, we propose the hypothesis that age-dependent alterations in condensate assembly, dynamics and material properties may contribute to oocyte aging by promoting defects in mitochondrial function, proteostasis and epigenetic regulation. Although attractive, this hypothesis requires further testing, and future studies should define the role of biomolecular condensates in reproductive aging.

## 3. Role of Apoptosis and Regulated Cell Death in Oocyte Aging

Regulated cell death pathways play a fundamental role in maintaining oocyte quality throughout the reproductive lifespan. By selectively eliminating damaged or developmentally compromised germ cells, these mechanisms prevent the accumulation of defective oocytes and contribute to the preservation of reproductive fitness.

In *C. elegans*, apoptosis functions as an active quality control system. Defective or supernumerary germ cells are eliminated, allowing resources to be redirected toward the maintenance of healthier oocytes. Disruption of apoptotic pathways leads to reduced oocyte quality and accelerated reproductive decline, highlighting the importance of programmed cell death in germline homeostasis [7,47]. In addition, apoptosis contributes to genome surveillance by removing oocytes carrying excessive DNA damage or unresolved recombination defects.

In contrast, apoptosis in vertebrate oocytes often represents a terminal response to accumulated cellular damage. In fish, postovulatory aging is accompanied by activation of pro-apoptotic pathways, increased caspase activity and progressive deterioration of oocyte quality [48]. Similar processes have been documented in other vertebrates and are frequently associated with mitochondrial dysfunction, oxidative stress and loss of developmental competence.

Importantly, apoptosis operates alongside other regulated cell death pathways. Autophagy, including selective forms such as mitophagy, contributes to the removal of damaged cellular components and can either promote cell survival or facilitate oocyte elimination under conditions of severe stress. The balance between these pathways appears to shift with age, influencing the fate and quality of individual oocytes.

From an evolutionary perspective, regulated cell death can be viewed as an integral component of oocyte quality control. Rather than simply removing damaged cells, apoptosis and autophagy help maintain the integrity of the germline by coordinating resource allocation, genome surveillance and organelle quality control. Their age-dependent dysregulation therefore contributes to the broader systems-level failure that characterises reproductive aging.

## 4. Epigenetic Alterations

Epigenetic drift, characterised by progressive alterations in chromatin organization and gene regulation, represents another conserved hallmark of oocyte aging. A prominent feature of this process is the loss of heterochromatin integrity, which can lead to the reactivation of transposable elements (TEs), genomic instability and reduced developmental competence [49,50].

In vertebrates, aging oocytes exhibit alterations in DNA methylation patterns, chromatin remodelling and transcriptional regulation. These changes are frequently accompanied by transposable element activation, DNA damage accumulation and impaired meiotic progression, all of which contribute to declining fertility and increased developmental abnormalities [51,52].

Compared with vertebrates, DNA methylation systems in insects are highly variable, ranging from extremely reduced or even absent in some lineages to functionally important in others. Consequently, many insects rely more heavily on alternative mechanisms of genome defence, among which the piRNA pathway plays a central role. In *D. melanogaster*, piRNAs guide PIWI proteins to transposable element transcripts, promoting both transcript degradation and heterochromatin formation at target loci [53,54]. This mechanism provides an effective alternative to extensive DNA methylation-based genome protection and highlights the evolutionary diversity of epigenetic maintenance strategies.

However, important exceptions occur among social insects. In honeybees (*Apis mellifera*), nutritional and environmental signals influence reproductive status through DNA methylation and other epigenetic modifications, generating profound differences between long-lived reproductive queens and short-lived sterile workers [55]. These observations illustrate the remarkable plasticity of epigenetic regulation in insects and suggest that different epigenetic mechanisms may contribute to the regulation of reproductive lifespan.

Taken together, these findings suggest that epigenetic alterations contribute substantially to reproductive aging by linking chromatin organization, transposon control and gene regulation. Because condensate-associated structures such as nuage participate in RNA-mediated genome defence and transposon silencing, age-dependent changes in condensate function may represent an important but still poorly understood driver of epigenetic instability in aging oocytes (Figure 5).

## 5. Ovarian Microenvironment and Evolutionary Adaptations

Oocyte aging is influenced not only by intrinsic cellular processes but also by the surrounding ovarian microenvironment. Across animal taxa, specialised supporting cells create a niche that provides nutrients, regulatory signals and quality control functions essential for maintaining oocyte integrity.

In insects with meroistic ovaries, developing oocytes are supported by nurse cells that transfer RNAs, proteins, organelles and other cytoplasmic components through intercellular bridges. This arrangement provides an efficient mechanism for resource allocation and contributes to mitochondrial quality control, as high-quality organelles are preferentially transported to the developing oocyte while damaged components are removed through apoptotic and autophagic pathways [27,56,57].

In mammals, analogous supportive functions are performed by granulosa cells, which maintain metabolic communication with the oocyte and regulate its development through bidirectional signalling.

Recent studies further emphasize that communication between the oocyte and its surrounding somatic cells is a dynamic, bidirectional process rather than a passive support system. Using *D. melanogaster* as an in vivo model, it was demonstrated that preovulatory follicle aging is regulated by reciprocal signalling between the oocyte and follicle cells [58]. The study revealed a positive feedback loop linking mitochondrial dysfunction in the oocyte with progressive functional decline of the surrounding somatic cells, while the stress-response protein Sestrin suppresses this degenerative circuit during the early stages of follicle aging [58]. These findings indicate that deterioration of the ovarian microenvironment may actively contribute to reproductive aging rather than simply representing a consequence of declining oocyte quality. Aging disrupts these interactions, leading to impaired metabolic support, increased oxidative stress and reduced capacity to maintain oocyte homeostasis [10,59]. Although these systems differ substantially in organization, both illustrate a common evolutionary principle: oocyte quality depends not only on autonomous cellular maintenance mechanisms but also on the integrity of the surrounding microenvironment. Age-related deterioration of this external network amplifies the effects of mitochondrial dysfunction, proteostasis collapse and genome instability within the oocyte itself. These observations highlight that oocyte aging is a multicellular process emerging from reciprocal interactions between the oocyte and its surrounding microenvironment. Despite profound differences in ovarian organization across animal taxa, maintenance of oocyte quality consistently depends on coordinated communication between germline and supporting cells. Evolution has therefore produced diverse strategies for protecting female germ cells, yet all ultimately face the same challenge: preserving oocyte quality over extended periods while minimizing the accumulation of cellular damage.

## 6. Conclusions

Oocyte aging is traditionally viewed through the lens of individual cellular defects, such as chromosomal instability, mitochondrial dysfunction, proteostasis collapse or epigenetic drift. Evidence from vertebrate and invertebrate models, however, suggests that these processes should not be considered in isolation. Instead, they might represent interconnected manifestations of a broader decline in the cellular quality control systems responsible for preserving oocyte integrity throughout prolonged periods of meiotic arrest. Diverse strategies that delay or mitigate reproductive aging can operate in various animal taxa (Table 1). Despite their diversity, these mechanisms converge on a common goal: maintaining genome stability, organelle functionality and proteome integrity within one of the longest-lived cell types in the animal body.

An interesting aspect emerging from recent studies is the growing appreciation of biomolecular organization during oogenesis. Structures such as the Balbiani body, nuage, ELVAs, ELYSAs and PPLC may be viewed as dynamic regulatory hubs that can potentially coordinate multiple aspects of oocyte homeostasis rather than as passive cellular compartments. We therefore propose the hypothesis that age-dependent alterations in the assembly, dynamics and material properties of biomolecular condensates may contribute to reproductive aging. Whether condensate dysfunction represents a cause, a consequence, or part of a reciprocal relationship with these processes remains to be established. This perspective provides a unifying framework that links previously disconnected observations across diverse animal models. At present, however, direct functional evidence remains limited, particularly outside a small number of well-established model organisms, and further comparative studies across diverse animal taxa will be essential to evaluate this hypothesis.

Future research should move beyond the analysis of individual aging pathways and focus on understanding how quality control networks interact within the oocyte. Particular attention should be given to the role of biomolecular condensates in coordinating these interactions, the mechanisms underlying their age-dependent remodeling and their potential as targets for preserving reproductive longevity. Comparative studies on both model and non-model organisms will be especially valuable, as they can reveal both conserved principles and evolutionary innovations in the maintenance of oocyte quality. Naturally long-lived species, such as the naked mole-rat, may prove particularly interesting in this context, allowing us to reveal evolutionary mechanisms that sustain oocyte quality and reproductive function throughout life [60]. Ultimately, understanding how oocytes preserve cellular order over time may provide broader insights into the fundamental biology of aging itself. Oocytes are among the longest-lived cells in the animal body, and their gradual decline offers a unique opportunity to investigate how complex cellular systems maintain functionality over extended timescales and why they eventually fail.

## Figures and Tables

**Table 1:** Comparative overview of the principal mechanisms contributing to oocyte aging across representative animal models and the current level of evidence supporting each process. Biomolecular condensates are presented separately as the conceptual framework proposed in the present review.

Age-Related Process/Hallmark	Representative Animal Model	Level of Evidence	References
Chromosomal instability and meiotic defects	human, mouse, *D. melanogaster*, *C. elegans*	+++	[4,14,15,16,17,18,22]
Mitochondrial dysfunction and oxidative stress	human, mouse, naked mole-rat, insects	+++	[25,26,27,28,29,32]
Proteostasis collapse	Mouse, *C. elegans*, insects	++	[35,36,37,39]
Epigenetic alterations	Human, honeybee, *D. melanogaster*	+++	[51,52,53,54,55]
Ovarian microenvironment	Mammals, insects	++	[27,56,57,58]
Biomolecular condensates	Vertebrates and invertebrates	+	hypothesis proposed in this review

+++ strong, ++ emerging, + proposed.

## Data Availability

No new data were created or analyzed in this study. Data sharing is not applicable to this article.
